# An Integrated Psychosocial Theory of Loneliness: A Multilevel Sociological and Psychological View

**DOI:** 10.1093/geroni/igaf122.3539

**Published:** 2025-12-31

**Authors:** Bianca Suanet, Marja Aartsen, Denis Gerstorf

**Affiliations:** Linköping University, Norrköping, Ostergotlands Lan, Sweden; Oslo Metropolitan University, Oslo, Oslo, Norway; Humboldt University Berlin, Humboldt University Berlin, Berlin, Germany

## Abstract

Loneliness is increasingly recognized as a pressing societal issue, yet theoretical integration across disciplines remains limited. While psychological research often focuses on individual factors, sociological approaches emphasize broader structural and cultural conditions. This article proposes an integrative framework that bridges micro-, meso-, and macro-level perspectives to better understand the complex, multi-layered nature of loneliness across the life course with a focus on later life. We synthesize insights from psychological and sociological perspectives, emphasizing how community, network, and institutional contexts shape individual experiences of loneliness. Building on existing conceptual models and empirical findings, we identify core contextual pathways and mechanisms that operate across levels. The proposed systems model outlines how loneliness emerges through dynamic interactions between societal structures, cultural norms, community settings, and individual factors. We emphasize key intersections between levels, including macro-meso (e.g., welfare policy and community resources), macro-micro (e.g., cultural norms and non-normative life transitions), and meso-micro (e.g., community characteristics and social support). The model supports a context-sensitive understanding of how structural inequalities and cultural norms affect loneliness. Our framework contributes to a more interdisciplinary and multi-level understanding of loneliness by linking structural, community, and individual processes. It provides a foundation for developing context-sensitive, equity-informed intervention elements that address loneliness not only through personal support, but also by transforming the social environments in which loneliness emerges, across different life stages and population groups.

